# Syphilitic Affections of the Heart and Aorta

**Published:** 1903-12-19

**Authors:** 


### SYPHILITIC AFFECTIONS OF THE HEART AND AORTA.

In an instructive paper recently read before the New York Post-graduate Clinical Society Dr. Leonard Weber[1] drew attention to the practical importance of recognising the influence of syphilis in causing disease of the heart and great vessels. Forty years ago the heart was thought to be practically uninfluenced by syphilis; then, thanks to the researches of Virchow, Heubner and others, it became known that syphilis was a rather frequent cause of arterial degeneration through sclero-gummatous processes, leading to endoarteritis obliterans with its baneful influence upon the nutrition of the parts supplied by the diseased vessel. Now it is recognised that sclerosis of the coronary arteries, and softening and degeneration of the cardiac muscle occurring in persons under 50 years of age, are often of syphilitic origin. In a paper published in the *Medical Record* in 1884, Dr. Weber showed that of 125 cases of syphilis treated by him and observed for a period of from 15 to 20 years there were but two who presented marked symptoms of specific disease of the brain (hemiplegia) and died of it, and that these two were men who, for one reason or another, could not be convinced that they had syphilis, and abandoned treatment during the first year of observation. Not one of the whole number had tabes or heart disease. It is probable therefore that efficient anti-syphilitic treatment applied early and extended over a long period will go far to prevent nervous or visceral lesions later on in life. It is in those cases where the early symptoms of specific disease have been slight and the patient has not been treated at all, or else only in a perfunctory manner, that late troubles are likely to arise. The actual lesions which may be found post-mortem are atheroma of the aorta, pulmonary artery, and coronary arteries with endoarteritis and periarteritis and interstitial myocarditis. In some cases inflammatory processes may be developed in and about the smaller vessels, leading to degeneration of the myocardium, when the greater vessels exhibit no signs of disease.

The symptoms may be those of angina pectoris alone, or there may be enlargement of the heart, weakness of the beat, accentuated second sound, arythmia, tachycardia, and dyspnœa on exertion. In the absence of a history or of signs of specific infection it will be almost impossible to arrive at a correct diagnosis in the early stages of the heart affection. In the absence of any other satisfactory etiology, such as rheumatism, alcohol, and chronic nicotine poisoning, to account for the above symptoms occurring in individuals below fifty years of age it is advisable to assume the cause to be syphilis, and try the effect of anti-syphilitic treatment.

Next to specific sclerosis of the coronary arteries and their branches, in frequency and practical importance must be placed syphilitic aortitis, with its sequelæ, degeneration of the semilunar valves and aneurysm. These affections of the aorta will be associated with cardiac hypertrophy and muscular degeneration also, particularly when the valves are much diseased. Out of a dozen cases of aortic aneurysm which Dr. Weber has had under his care, syphilis had been present in ten. The treatment in all cases consists of the administration of mercury and iodides with a view to arresting if possible any further development of the disease process. Even old-standing cases may sometimes be much improved by specific treatment.

[1] Post-Graduate, December, 1903.

